# miRNA-target gene network analysis in siblings with cystic fibrosis and phenotypic variability

**DOI:** 10.55730/1300-0144.6054

**Published:** 2025-04-28

**Authors:** Ayberk MUSTAFAOĞLU, Senem NOYAN, Yeliz Z. AKKAYA ULUM, Bala GÜR DEDEOĞLU, Nagehan EMİRALİOĞLU, Uğur ÖZÇELİK, Ebru YALÇIN, Deniz DOĞRU, Nural KİPER, Didem DAYANGAÇ ERDEN

**Affiliations:** 1 Faculty of Medicine, Hacettepe University, Ankara, Turkiye; 2 Biotechnology Institute, Ankara University, Ankara, Turkiye; 3 Department of Medical Biology, Faculty of Medicine, Hacettepe University, Ankara, Turkiye; 4 Department of Pediatric Pulmonology, Faculty of Medicine, Hacettepe University, Ankara, Turkiye

**Keywords:** Cystic fibrosis, genotype phenotype correlation, phenotypic variability, miR-449c-5p, inflammation

## Abstract

**Background/aim:**

Cystic fibrosis (CF) is an autosomal recessive disease caused by mutations in the cystic fibrosis transmembrane conductance regulator (*CFTR*) gene. CF is characterized by respiratory tract infections, pancreatic insufficiency, meconium ileus, intestinal obstruction, and male infertility. A genotype to phenotype correlation is difficult to establish because of the heterogeneity of disease severity. Even patients with the same *CFTR* mutation can have varying clinical severities. In recent years, studies have explored the role of microRNA (miRNA) expression in the regulation of respiratory diseases. However, no research has been conducted to date on miRNAs in siblings with the same *CFTR* mutation.

**Materials and methods:**

Nasal cells of CF siblings from two families with discordant phenotype (n = 2 per family) were collected, and differentially expressed miRNAs were identified using miRNA arrays. Differentially expressed miRNAs and their target genes were determined using several bioinformatic databases and tools.

**Results:**

miR-449c-5p, miR-92b-3p, miR-34c-3p, miR-34c-5p, miR-6732-5p, and miR-4793-3p were differentially expressed in patients with severe disease compared to mild. *CXCL1*, *CXCL2*, *DUSP1*, *GCLC*, *ICAM1*, *KIT*, *PRKAA2*, and *PTGS2* genes were identified as the target genes of candidate miRNAs (miR-34c-3p, miR-92b-3p, miR-449c-5p, miR-4793-3p). miRNA-mRNA interaction network analysis was performed and strong interaction was shown between miR-449c-5p target genes (*CXCL1*, *CXCL2*, *PTGS2*, *ICAM1*). *CXCL1* expression decreased 5.28-fold in patients with severe disease compared to those with mild (p = 0.01).

**Conclusion:**

Our results highlight the importance of miR-449c-5p interaction with *CXCL1* and other target genes related to inflammation. Further studies should focus on the functional analysis of miR-449c-5p.

## 1. Introduction

Cystic fibrosis (CF) is an autosomal recessive genetic disease caused by mutations in the CF transmembrane conductance regulator (*CFTR*) gene on 7q31.2. The incidence rate of CF is approximately 1 in 3000 in Türkiye [1–3]. *CFTR* encodes a chloride channel located on the apical cell membrane that is regulated by cyclic AMP (cAMP)-dependent phosphorylation. The lungs, sinuses, sweat glands, liver, pancreas, intestines, and male reproductive tract are the major organs affected by mutations in the *CFTR* gene [4]. Abnormal function of ion channels causes ineffective Cl^−^ and HCO_3_^−^ secretion, and subsequent dysregulation of ENaC-mediated Na^+^ transport causes dehydration in lung airways. Bacterial colonization and lung damage can occur because of the static mucus on the airway surface [5]. Chronic lung infection by *Staphylococcus aureus* and *Pseudomonas aeruginosa* increases mortality and morbidity of CF patients [6,7]. In addition to classical symptoms, defective CFTR protein causes CF-related diabetes (CFRD) and cirrhosis [8,9]. More than 2000 mutations have been identified in the *CFTR* gene [10]. Mutations have been classified into six major groups according to their cellular phenotype: Class IA and IB (loss of function), Class II (affected trafficking), Class III (reduced gating), Class IV (decreased conductance), Class V (insufficient function), and Class VI (reduced stability) [11]. In Türkiye, mutations are heterogeneous and differ from those in European populations. The most common mutation is F508del with an allelic frequency of 28%. Other common mutations are N1303K (4.9%), G542X (4.5%), 1677delTA (4%), and G85E (3.8%) [12].

Although CF is caused by mutations in the *CFTR* gene, patients with the same genotype may show variability in clinical severity and prognosis of the disease. Many studies have focused on modifier genes [13–15] and transcriptomic approaches [13,16–19]. microRNAs (miRNAs) are small noncoding RNAs, 20–24 nucleotides in length. They regulate gene expression posttranscriptionally and are involved in various biological processes including differentiation, apoptosis, and inflammation [20]. Through binding to the 3′ untranslated region (UTR), they can either enhance or suppress the expression of target messenger RNAs (mRNA) [21–23]. In recent years, studies on miRNA in bronchial cell lines or primary cell cultures have indicated that changes in miRNA expression may be related to CF disease progression and severity [16,24–26]. To date, no studies have examined miRNA expression in nasal epithelial cells obtained from siblings with the same genetic background but different severity of CF. In this study, differentially expressed miRNAs in nasal epithelial cells from CF siblings were identified for the first time. Target genes of candidate miRNAs and related pathways were determined by integrating different transcriptome platform assays related to CF pathogenesis.

## 2. Materials and methods

### 2.1. Patients

This study was conducted with four CF patients from two families (family 1: F1-P1, F1-P2, family 2: F2-P1, F2-P2). The clinical severity of siblings from both families was determined by predicted percentage of forced expiratory (FEV1%), hepatic involvement, CFRD, and recurrent lung infection parameters. All patients had Class II mutations in the *CFTR* gene.

F1-P1 was 12 years old when the sample was collected and had mild hepatic involvement compared to his sister. The predicted FEV% at 1 s was 88. Sweat chloride concentration was 96 mmol/L. He had no recurrent lung infection. On the other hand, F1-P2 was 16 years old and had severe hepatic involvement with cirrhosis and CFRD. Predicted FEV1% was 105. Sweat chloride concentration was 110 mmol/L. She had no recurrent lung infection. Both siblings harbored the F508del/G85E mutation.

F2-P1 was 6 years old when the sample was collected and had mild hepatic involvement compared to his sister. Sweat chloride concentration was 101 mmol/L. He had recurrent lung infection. On the other hand, F2-P2 was 3 years old and had severe hepatic involvement. Sweat chloride concentration was 96 mmol/L. She had progressive recurrent lung infection. Both siblings were homozygous for the F508del mutation. The detailed clinical parameters of siblings are described in Table 1.

### 2.2. RNA isolation and microarray analysis

Nasal respiratory epithelial cell samples were obtained from patients who did not have any airway infection in the last 14 days. Total RNA was isolated from patients using miRCURY RNA Isolation Kit (Exiqon, Vedbæk, Denmark). The quality and quantity of RNA were determined using a NanoDrop ND 1000 spectrophotometer (Thermo Scientific, Waltham, MA, USA). GeneChip miRNA 4.0 Array (Affymetrix, Santa Clara, CA, USA) was used to determine expression levels of miRNAs according to the manufacturer’s recommendations. As starting material for microarray analysis, 900 ng total RNA was used. As quality control criteria for the miRNA array experiment, signal intensities (presence/absence values) and signal histograms of all chips were evaluated. The raw data was analyzed using Affymetrix Transcriptome Analysis Console version 4 (TAC 4.0). The expression profiles of patients with severe disease (F1-P2 and F2-P2) were compared with those with mild disease (F1-P1 and F2-P1) to obtain p-values and fold changes for each miRNA. Differentially expressed miRNAs with statistically significant differences (p < 0.05) and fold changes ≥ 2.0 were listed.

### 2.3. Bioinformatic and statistical analyses

#### 2.3.1. Target gene analysis

The target genes of differentially expressed candidate miRNAs were detected with miRWalk version 3.0 and TargetScan version 8.0 databases. miRWalk 3.0 (http://mirwalk.umm.uni-hd.de/) is an miRNA-mRNA prediction tool that incorporates data from the TarPmiR database. It uses a random forest-based approach to predict miRNA target sites and utilizes features such as miRNA seed-matching sites, folding energy of target sites, and AU-rich elements (AREs). In version 3, the database focuses on a machine learning approach and utilizes three third-party databases: miRTarBase, miRDB, and TargetScan. miRTarBase provides validated gene targets reported in the literature, while the other databases offer predicted target genes.

TargetScan 8.0 (https://www.targetscan.org/) predicts miRNA targets by identifying conserved 8mer, 7mer, and 6mer binding sites for each miRNA. Predictions are ranked by different parameters such as context++ scores of the sites and probability of conserved targeting.

We determined the target gene lists through miRWalk version 3.0 and TargetScan version 8.0 databases.

#### 2.3.2. Gene ontology (GO) enrichment analysis

The gene lists obtained from miRWalk version 3.0 and TargetScan version 8.0 databases for six miRNAs were classified using the gene set enrichment analysis tool Enrichr ( https://maayanlab.cloud/Enrichr/ ). Enrichr contains gene set libraries such as HumanCyc, PANTHER, KEGG, Reactome, and GO Biological Process. It uses p-value, q-value, rank (Z-score), and combined scores. GO enrichment analysis for biological processes was performed with target genes of each candidate miRNA using the Enrichr platform.

#### 2.3.3. Intersection of target genes of differentially expressed miRNAs and CF array

The RT^2^ Profiler PCR Array for human cystic fibrosis (Qiagen, Hilden, Germany) consists of 84 genes related to CF and CF-related pathways such as immune and inflammatory response, neutrophil chemotaxis, and ion transport. In a previous study, the same RNA isolated from the patients was run using the PCR array. The severe and mild siblings were compared and significantly differentially expressed genes were determined. Data obtained from the siblings were reanalyzed and intersection analysis was performed between genes obtained from the RT^2^ Profiler PCR Array and target genes of miR-34c-3p, miR-34c-5p, miR-92b-3p, miR-449c-5p, miR-4793-3p, miR-6732-5p using VENNY version 2.1.0 ( https://bioinfogp.cnb.csic.es/tools/venny/ ).

#### 2.3.4. Statistical analysis

For target gene analysis of differentially expressed miRNAs, relative quantitation (2^−ΔCt^) was used to calculate the results by comparing the severe and mild groups. The results were evaluated using Student’s t-test, where p < 0.05 was considered significant. Statistical analysis was performed and graphics were plotted using GraphPad Prism version 8.3.0 ( https://www.graphpad.com/ ).

## 3. Results

### 3.1. Differential expression of six miRNAs in patients with severe versus mild disease

Using TAC 4.0, miRNA array analysis was conducted to compare patients with severe (F1-P2 and F2-P2) and mild (F1-P1 and F2-P1) disease. Six differentially expressed miRNAs with at least a 2-fold change and p < 0.05 were identified as either upregulated or downregulated: miR-449c-5p (FC = 54.69), miR-92b-5p (FC = 24.6), miR-34c-3p (FC = 16.22), miR-34c-3p (FC = 4.44), miR-6732-5p (FC = −6.25), and miR-4793-3p (FC = −6.53) (Table 2).

**Figure 1:**
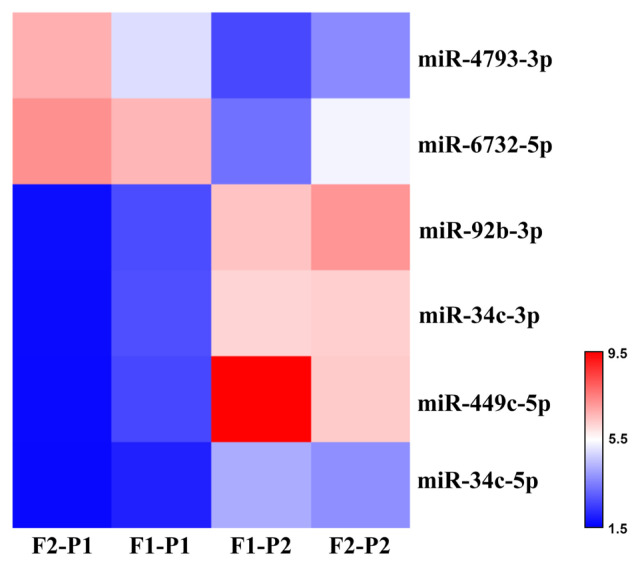
Heat map of differentially expressed miRNAs. Each vertical column represents patients (F2-P1 and F1-P1: mild, F1-P2 and F2-P2: severe) and horizontal rows represent miRNAs. Red to blue colors represent higher to lower fold change values.

A heat map was generated using TAC 4.0 based on the expression values of the differentially expressed miRNAs (Figure 1). Patients with severe or mild disease were divided into two groups according to the expression profiles of the six differentially expressed miRNAs. The expression levels of four miRNAs (miR-92b-3p, miR-34c-3p, miR-449c-5p, and miR-34c-5p) were higher in the severe group compared to mild patients, whereas the expression levels of two miRNAs (miR-4793-3p and miR-6732-5p) were lower in severe patients.

**Figure 2:**
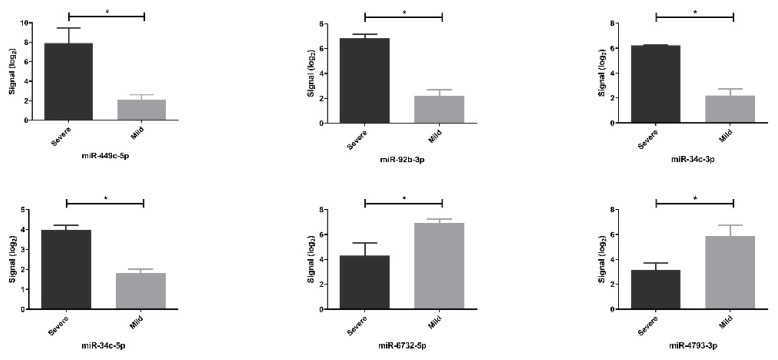
Raw array intensity of samples for each miRNA. Unpaired Student’s t-test was performed between severe and mild siblings (p < 0.05).

The sample signals for each miRNA were calculated, and the results are shown using TAC 4.0 in Figure 2.

### 3.2. Target gene identification of candidate miRNAs via miRWalk and TargetScan

The target genes of candidate miRNAs were identified using the miRWalk version 3.0 database, while the predicted target genes were identified using TargetScan and miRDB via miRWalk. The validated target genes were obtained from miRTarBase using the miRWalk software (Table 3a).

Among miRNA analysis tools, TargetScan version 8.0 is one of the most common and widely preferred by researchers. Target genes could not be identified for the four candidate miRNAs (miR-34c-3p, miR-449c-5p, miR-4793-3p, and miR-6732-5p) using the TargetScan tool in miRWalk version 3.0, possibly because miRWalk version 3.0 only labels TargetScan version 8.0 results from conserved sites. Therefore, nonconserved sites were also analyzed. miRWalk version 3.0 utilizes data from the TarPmiR database, including both conserved and nonconserved sites, for determining the target genes. The number of target genes for these four miRNAs was greater than zero. Target gene data obtained from Targetscan version 8.0 were also included. The number of target genes are listed in Table 3b. Target gene lists obtained from TargetScan version 8.0 and miRWalk version 3.0 were used for GO enrichment analysis.

### 3.3. GO enrichment analysis of target genes

**Figure 3:**
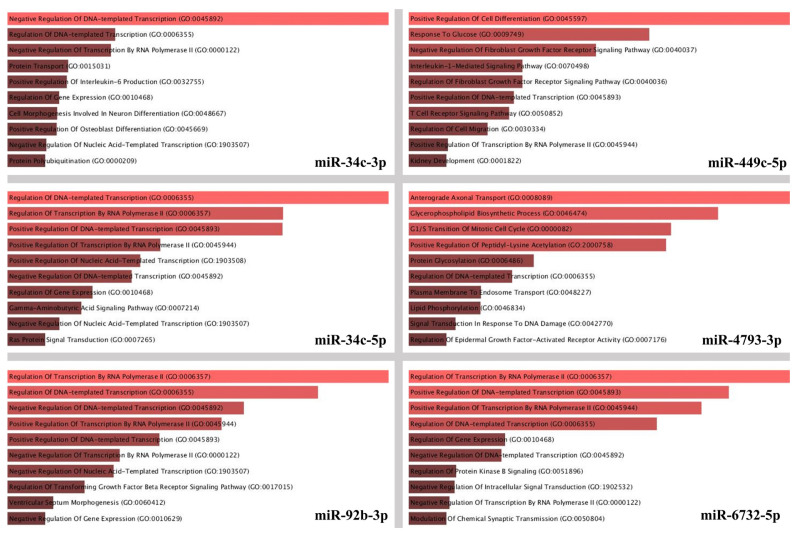
GO enrichment analysis of the target genes of miR-34c-3p, miR-449c-5p, miR-34c-5p, miR-4793-3p, miR-92b-3p, and miR-6732-5p was performed against the biological processes gene set. Colored bar graphs are sorted by p-value (p < 0.05). The longer and light colored bars represent the GO terms that are more significant.

Target genes of the candidate miRNAs were subjected to GO enrichment analysis against biological process gene sets (Figure 3). The top four significantly enriched GO terms for miR-449c-5p were regulation of cell differentiation, response to glucose, regulation of fibroblast growth factor receptor signaling, and interleukin-1 mediated signaling. The miR-4793-3p GO terms are mainly related to protein transport, glycosylation, acetylation, and glycerophospholipid biosynthesis. miR-34c-5p, miR-34c-3p, miR-6732-5p, and miR-92b-3p target genes were enriched for biological processes related to transcription regulation.

### 3.4. Common genes from the intersection of target genes from the miRNA and CF array

The intersection of the genes from the RT^2^ Profiler PCR Array for human cystic fibrosis and the target genes of each miRNA was evaluated. The genes common to each miRNA were also identified. *KIT*, *GCLC*, and *PRKAA2* were upregulated, whereas *CXCL1*, *CXCL2*, *ICAM1*, *DUSP1*, and *PTGS2* were downregulated (Table 4).

**Figure 4:**
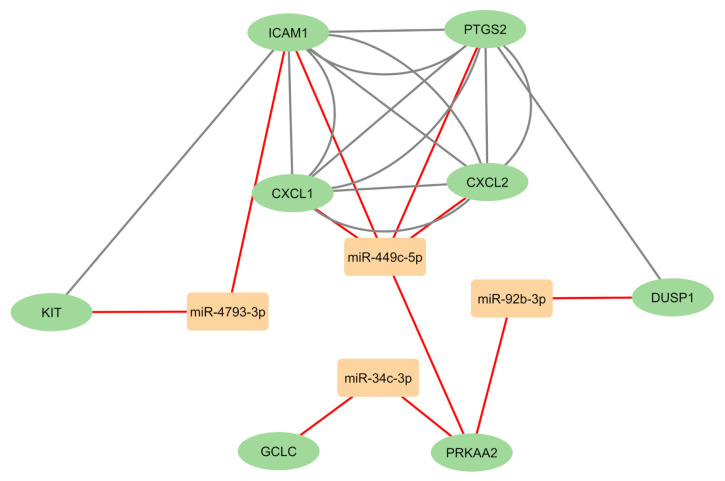
Proposed model of miRNA-mRNA interaction network in CF severity visualized with Cytoscape 3.9.1. Orange boxes represent candidate miRNAs and green ellipse represent target genes of miRNAs. Red lines show miRNA-target gene interaction for each miRNA. Grey lines show gene-gene interactions. STRING version 10.5 was used to determine interactions between target genes.

Using the common genes obtained from the intersection analysis (*CXCL1*, *CXCL2*, *DUSP1*, *GCLC*, *ICAM1*, *KIT*, *PRKAA2*, and *PTGS2*) and the differentially expressed miRNAs (miR-34c-3p, miR-92b-3p, miR-449c-5p, and miR-4793-3p), an miRNA-mRNA interaction network analysis was performed using STRING version 10.5 and visualized in Cytoscape version 3.91 (Figure 4). The results highlighted the interaction of miR-449c-5p with *CXCL1*, *CXCL2*, *PTGS2*, and *ICAM1*. Additionally, *PRKAA2* was a common target of three differentially expressed miRNAs: miR-34c-3p, miR-92b-3p, and miR-449c-5p.

**Figure 5:**
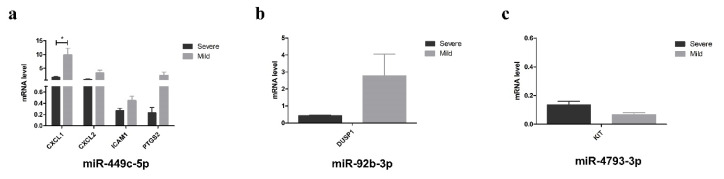
The expression values of genes that are targets of (a) miR-449c-5p, (b) miR-92b-3p, and (c) miR-4793-3p obtained from RT^0^ Profiler PCR Array for human cystic fibrosis. Unpaired Student’s t-test analysis was performed comparing the severe group to the mild group. *KIT* was upregulated in the severe group (p = 0.07). *CXCL1* (p = 0.01), *CXCL2* (p = 0.05), *ICAM1* (p = 0.07), *DUSP1*, (p = 0.08), and *PTGS2* (p = 0.08) were downregulated. The downregulation of CXCL1 gene expression was found statistically significant.

Target gene validation revealed that *CXCL1*, *CXCL2*, *ICAM1*, and *PTGS2* were targets of miR-449c-5p. *DUSP1* and *KIT* were identified as miR-92b-3p and miR-4793-3p targets, respectively. The results of the target gene analysis are presented in Table 5 and Figure 5. *CXCL1* expression considerably decreased by 5.28-fold in patients with severe disease compared to patients with mild disease (p = 0.01). In patients with severe disease, *KIT* (FC = 2.10) expression increased, whereas *ICAM1* (FC = −1.65), *CXCL2* (FC = −3.20), *DUSP1* (FC =−4.10), and *PTGS2* (FC = −10.36) expression decreased, although the differences were not statistically significant.

In this study, we demonstrated the relationship between miRNAs and the phenotypic variability observed in patients with the same mutation. Differentially expressed miRNAs were determined in severe CF patients compared to mild patients. The miRNA-mRNA interaction network analysis showed strong interactions between miR-449c-5p and its target genes (*CXCL1*, *CXCL2*, *PTGS2*, and *ICAM1*)—genes involved in inflammation related pathways. Our preliminary results pave the way for functional analysis of miR-449c-5p. Also a prognostic miRNA signature can potentially facilitate the early determination of the course of the disease.

## 4. Discussion

Although monogenic disorders are caused by mutations in a single gene, phenotypic heterogeneity can be observed in disease pathogenesis. In CF, a multisystem disorder, establishing genotype to phenotype correlations is particularly difficult. Studies regarding patients with the same *CFTR* mutation but different clinical symptoms have suggested that miRNAs are involved in determining the clinical variability and prognosis of the disease.

Altered miRNA expression can directly or indirectly affect the expression level of the *CFTR* gene. Megiorni et al. [24] showed that miR-101, and miR-494 directly suppress *CFTR* expression, whereas another study indicated that miR-138 indirectly downregulates *CFTR* expression by targeting the transcriptional repressor gene switch-independent 3 homolog A (*SIN3A*) [25]. Differentially expressed miRNAs are potentially critical in regulating the expression of proinflammatory mediators and immune response-related genes important for CF progression. Luly et al. [16] showed that in CF macrophages, the inhibition of miR-146a led to an increase in proinflammatory cytokine interleukin-6 (IL-6) production. Increased miR-17 and miR-93 levels are also associated with decreased production of interleukin-8 (IL-8), another principal cytokine in immune and inflammatory responses. The expression level of IL-8 is also indirectly regulated by miR-155. The upregulation of miR-155 in CF cells results in SH-2 containing inositol 5′ polyphosphatase 1 (*SHIP1*) gene translation inhibition, which leads to the activation of the phosphatidylinositol-3 kinase/protein kinase B (PI3K/AKT) related signaling pathway. Consequently, this results in IL-8 secretion. In addition to inflammation, Tsuchiya et al. [15] demonstrated that miR-155 could also trigger fibrosis by suppressing regulatory associated protein of mTOR complex 1 (RPTOR) expression. In contrast to the aforementioned miRNAs, miR-126 is antiinflammatory in CF. Oglesby et al. [13] reported decreased miR-126 expression in CF airway epithelial cells. miR-126 downregulation is related to target of Myb1 membrane trafficking protein (TOM1) upregulation, which is involved in the TLR2 and TLR4 signaling pathways.

miRNAs can also be used as biomarkers of CF. Cook et al. [27] reported that miR-21, miR-25, and miR-122 could be used to identify patients with CF and liver disease. miR-21, miR-25, and miR-122 levels are upregulated in patients with CF and liver fibrosis.

To date, no miRNA array has been performed with nasal samples obtained from discordant siblings from families of the same genetic origin. In this study, nasal respiratory epithelial cells that represent the lower respiratory tract were used to determine for the first time the miRNA profiles of patients with severe (F1-P2 and F2-P2) and mild (F1-P1 and F2-P1) disease with the same mutation.

In patients with severe disease, miR-449c-5p, miR-92b-5p, miR-34c-3p, miR-34c-5p, miR-6732-5p, and miR-4793-3p were compared to patients with mild disease. Target gene analysis of miRNAs was performed using miRWalk version 3.0 and TargetScan version 8.0. miRWalk version 3.0 ( http://mirwalk.umm.uni-hd.de/ ) uses machine learning-based algorithms and includes the TargetScan, miRDB, and miRTarBase datasets. miRTarBase covers experimentally validated target genes, whereas TargetScan and miRDB are databases for predicted miRNA target genes. In addition to the TargetScan dataset, which is included in miRWalk 3.0, the TargetScan version 8.0, a web tool (http://targetscan.org/vert_80/), was also used. This database contains information on the conservation of miRNA-binding sites. We compared the number of target genes obtained from both the TargetScan version 8.0 web tool and the TargetScan database, which is included in miRWalk version 3.0, and found that these databases showed different numbers of target genes. The miRWalk version 3.0 database uses gene datasets with conserved sites only and does not include nonconserved sites that were obtained from the TargetScan version 8.0 web tool. In humans, although many predicted sites exist, not all are conserved (Sticht, personal communication, June 6, 2023). Therefore, the number of target genes in TargetScan included in miRWalk version 3.0 was fewer than that in the TargetScan version 8.0 web tool itself. Another reason for obtaining a high number of target genes in TargetScan version 8.0 is that this web tool predicts the targets of miRNAs by checking for conserved 8mer, 7mer, and 6mer sites that match the seed area for each miRNA. Predictions using nonconserved miRNAs and weakly conserved sites are also available as alternatives. These alternatives also increase the total number of predicted target genes.

From the intersection of target genes of differentially expressed miRNAs obtained from the miRNA array and genes from the CF array, eight common genes (*CXCL1*, *CXCL2*, *DUSP1*, *GCLC*, *ICAM1*, *KIT*, *PRKAA2*, and *PTGS2*) were found. Target genes of miR-449c-5p (*CXCL1*, *CXCL2*, *ICAM1*, and *PTGS2*), miR-92b-3p (*DUSP1*), and miR-4793-3p (*KIT*) were validated by RT-qPCR using a CF array. Target genes were involved in inflammation-related pathways such as the chemokine-mediated signaling pathway, cellular response to chemokines, neutrophil chemotaxis, granulocyte chemotaxis, and neutrophil migration. Only *CXCL1* expression, which is a miR-449c-5p target gene, was significantly decreased in the severe group of patients compared to the mild group. *CXCL1* regulates granulopoiesis, neutrophil recruitment, and mobilization. Moreover, it induces neutrophil influx during bacterial infections. Keown et al. [28] noted that CF patients cannot mount effective host defense despite the abundance of neutrophils in the airways. Therefore, we suggest that the downregulation of chemoattractant and adhesion factors—such as *CXCL1*, *CXCL2*, *ICAM1*, and *PTGS2*—that strongly interact with miR-449c-5p may lead to impaired neutrophil function or abnormalities in proinflammatory stimuli.

Previous studies have shown that miR-34c-3p, miR-34c-5p, miR-92b-3p, miR-4793-3p, and miR-6732-5p are associated with several cancers, including colon, prostate, gastric, and pancreatic cancers. Meanwhile, miR-449c-5p is associated with pulmonary diseases. Cerón-Pisa et al. [29] found that miR-449c-5p was upregulated in chronic obstructive pulmonary disease (COPD) for both smokers and nonsmokers and concluded that miR-449c-5p could be a potential biomarker of COPD.

Identifying siblings with the same mutation but varying clinical severity is challenging for rare diseases. Despite the small sample size, this was the first miRNA analysis conducted on nasal samples from CF-discordant siblings of the same genetic origin. Our preliminary results indicate the importance of miR-449c-5p interaction with *CXCL1* and other target genes related to inflammation. In future studies, we plan to increase the number of patients and perform validation experiments on miRNA expression. Additionally, by conducting further in vitro functional analyses, the effects of miR-449c-5p can be evaluated and its direct target genes can be identified.

## Figures and Tables

**Table 1:** Genetic and clinical parameters of discordant siblings.

	Mutation type	Genotype	Sex	Age at sample collection (years)	Sweat chloride concentration	FEV1 (%predicted)	Malabsorption	Recurrent lung infection	Comorbidity	Disease severity
**Family 1**										
Patient 1 (F1-P1)	Class II	F508del/G85E	Male	12	96	88	Yes	No	Mild hepatic involvement	Mild
Patient 2 (F1-P2)	Class II	F508del/G85E	Female	16	110	105	Yes	No	Severe hepatic involvement with cirrhosis, CFRD	Severe
**Family 2**										
Patient 1 (F2-P1)	Class II	F508del/F508del	Male	6	101	-	Yes	Yes	Mild hepatic involvement	Mild
Patient 2 (F2-P2)	Class II	F508del/F508del	Female	3	96	-	Yes	Yes, progressive	Severe hepatic involvement	Severe

Abbreviations: CFRD: CF-related diabetes, FEV1: forced expiratory volume in 1 s.

**Table 2:** The list of differentially expressed miRNAs in severe compared to mild patients.

miRNA	Fold change (FC)	p-value
miR-449c-5p	54.69	0.0197
miR-92b-3p	24.6	0.0020
miR-34c-3p	16.22	0.0021
miR-34c-5p	4.44	0.0023
miR-6732-5p	−6.25	0.0495
miR-4793-3p	−6.53	0.0437

**Table 3a:** The number of target genes obtained from the miRWalk version 3.0 database.

miRNA	TargetScan	MiRDB	MirTarBase	TargetScan 8.0
miR-34c-3p	0	125	24	3132
miR-34c-5p	450	479	93	754
miR-92b-3p	477	427	334	1041
miR-449c-5p	0	283	63	4103
miR-4793-3p	0	118	120	5003
miR-6732-5p	0	133	62	2855

**Table 3b:** The number of target genes obtained from the TargetScan version 8.0 and TargetScan in miRWalk version 3.0 databases.

miRNA	TargetScan 8.0	TargetScan (miRWalk 3.0)
miR-34c-3p	3132	1052
miR-34c-5p	754	450
miR-92b-3p	1041	477
miR-449c-5p	4103	1867
miR-4793-3p	5003	2572
miR-6732-5p	2855	1697

**Table 4:** The common genes obtained from the intersection of target genes of miRNA array and CF array.

Genes	Description	Fold change	p-value
**Upregulated genes**			
*KIT*	V-kit Hardy-Zuckerman 4 feline sarcoma viral oncogene homolog	2.10	0.07
*GCLC*	Glutamate-cysteine ligase, catalytic subunit	1.67	0.07
*PRKAA2*	Protein kinase, AMP-activated, alpha 2 catalytic subunit	1.53	0.06
**Downregulated genes**			
*ICAM1*	Intercellular adhesion molecule 1	−1.65	0.07
*CXCL2*	Chemokine (C-X-C motif) ligand 2	−3.20	0.05
*DUSP1*	Dual specificity phosphatase 1	−4.10	0.08
*CXCL1*	Chemokine (C-X-C motif) ligand 1 (melanoma growth stimulating activity, alpha)	−5.28	**0.01**
*PTGS2*	Prostaglandin-endoperoxide synthase 2 (prostaglandin G/H synthase and cyclooxygenase)	−10.36	0.08

The downregulation of CXCL1 gene expression was statistically significant and is indicated in bold.

**Table 5:** The target genes of up- and downregulated miRNAs.

miRNA	Target genes
**Downregulated miRNA**	**Upregulated gene**
miR-4793-3p	*KIT*
**Upregulated miRNAs**	**Downregulated genes**
miR-449c-5p	*CXCL1*
	*CXCL2*
	*ICAM1*
	*PTGS2*
miR-92b-3p	*DUSP1*
